# Sulforaphane Determination by GC-MS Analysis: Mitigation of Thermal Degradation and Quality Evaluation of Two Broccoli-Based Supplements

**DOI:** 10.3390/molecules31152598

**Published:** 2026-07-25

**Authors:** Valentina Melini, Irene Baiamonte, Francesca Masciola, Antonio Raffo, Gina Rosalinda De Nicola

**Affiliations:** 1 Consiglio per la Ricerca in Agricoltura e l’Analisi dell’Economia Agraria (CREA), Centro di Ricerca Alimenti e Nutrizione, Via Ardeatina, 546, 00178 Roma, Italy; valentina.melini@crea.gov.it (V.M.); irene.baiamonte@crea.gov.it (I.B.); francesca.masciola@crea.gov.it (F.M.); 2 Consiglio per la Ricerca in Agricoltura e l’Analisi dell’Economia Agraria (CREA), Centro di Ricerca Orticoltura e Florovivaismo, Via dei Fiori, 8, 51017 Pescia, Italy

**Keywords:** glucoraphanin, glucosinolate, 3-butenyl isothiocyanate, dietary supplements, brassica vegetables

## Abstract

Sulforaphane (SF), the renowned hydrolysis product of glucoraphanin from broccoli, is known to undergo thermal degradation to 3-butenyl isothiocyanate (3-BITC) during gas chromatography–mass spectrometry (GC-MS) analysis. In this study, the effects on SF degradation associated with exposure to oven temperature during column transit, passage through the split/splitless injector, and through the transfer line to the mass spectrometer were investigated. Massive degradation was observed during column transit when the oven temperature exceeded 150 °C. Degradation during the passage through the injector increased with temperature, from 150 °C to 250 °C, and with decreasing split ratio, from 100:1 to 20:1. Conventional splitless conditions with the injector temperature set at 250 °C resulted in a degradation > 60%. Operating the GC-MS system with oven temperature below 150 °C prior to SF elution, an injector at 225 °C and a split ratio of 20:1, SF degradation was reduced to less than 1%. Under these optimized analytical conditions, the concentration of free SF in two broccoli-based dietary supplements was estimated based on external calibration and using 2,6-dimethylphenyl isothiocyanate as an internal standard. The GC-MS method proved to be a valuable tool for the quality evaluation of SF-labeled supplements integrated with the official EU ISO 9167:2019 HPLC-PDA glucosinolates analysis.

## 1. Introduction

**Figure 1:**
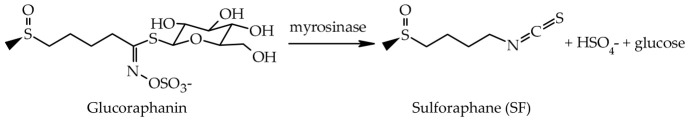
Reaction of myrosinase (*β*-thioglucoside glucohydrolase; EC 3.2.1.147) catalyzed hydrolysis of naturally occurring (*Rs*)-4-(methylsulfinyl)butyl glucosinolate (glucoraphanin) to produce (*Rs*)-4-(methylsulfinyl)butyl isothiocyanate (sulforaphane; SF).

Dietary sulforaphane ((*Rs*)-4-(methylsulfinyl)butyl isothiocyanate, SF) is one of the most extensively investigated bioactive isothiocyanates (ITCs) and a recognized health-promoting agent [1]. SF is not produced as such by the plant but rather released by the enzymatic action of myrosinase (*β*-thioglucoside glucohydrolase; EC 3.2.1.147) on the precursor glucosinolate (GSL) glucoraphanin ((*Rs*)-4-(methylsulfinyl)butyl GSL, GRA) (Figure 1) naturally occurring in Brassicaceae vegetables of significant economic importance and globally cultivated broccoli, cabbage, kale, and cauliflower [2,3,4].

A large body of evidence suggests a wide range of biological activities including antioxidant, anti-inflammatory, antimicrobial, neuroprotective, cardioprotective, and chemopreventive effects associated with SF intake [5,6,7]. Particular attention has been devoted to its ability to activate the nuclear factor erythroid 2-related factor 2 (Nrf2) pathway, modulate phase II detoxification enzymes, and regulate cellular responses against oxidative stress and inflammation [8]. These mechanisms have led to extensive investigations of SF in relation to cancer prevention, metabolic disorders, cardiovascular diseases, and neurodegenerative pathologies.

Broccoli-based dietary supplements have become popular and are marketed all over the world. Their preparations mainly consist of dried vegetables, either broccoli or broccoli sprouts, or extracts of broccoli seeds or sprouts. Depending on the composition and formulation of vegetable materials, they might contain glucoraphanin, SF, or glucoraphanin with added active myrosinase [9,10]. However, the definition of broccoli-based dietary supplements quality is critical. An accurate label should disclose the plant source with the variety name, as well as the actual glucoraphanin or SF titer. Marketing labels are instead not always straightforward about broccoli-derived ingredients. Often, the two components glucoraphanin and SF are invariably used without distinction, or even worse, the fancy name “sulforaphane glucosinolate” is used. Better industry monitoring and regulation are mandatory, and robust quality control is essential to enhance the consumer’s ability to make direct health span purchasing choices [10].

Because of its nutraceutical and pharmacological relevance, the accurate determination of SF in food matrices has become increasingly important for food quality assessment, nutritional studies, and the development of functional foods and nutraceutical products. However, SF determination remains analytically challenging due to its chemical instability, relatively high reactivity, and susceptibility to degradation induced by several parameters, including temperature, pH value, light exposure, oxidation, and sample preparation procedures [11,12]. Therefore, considerable efforts have been devoted to the development of sensitive, selective, and reproducible analytical methodologies suitable for its identification and quantification in complex matrices.

Currently, liquid chromatography-based methods are considered the analytical techniques of choice for SF determination. High-performance liquid chromatography (HPLC) and ultra-high-performance liquid chromatography (UHPLC), often coupled with diode-array detection (DAD) or tandem mass spectrometry (LC-MS/MS), are widely employed because they provide high sensitivity, selectivity, and robustness while minimizing thermal degradation phenomena [13]. In recent years, advanced LC-MS methodologies have enabled highly accurate quantification of SF and GSLs in broccoli and other Brassica vegetables, even at trace concentration levels and in highly complex matrices [14]. Moreover, high-resolution mass spectrometric approaches have improved analyte identification and structural characterization, contributing to a better understanding of SF occurrence in food products [15].

Nevertheless, the moderate volatility of this molecule also makes its separation by gas chromatography (GC) and its determination by GC-MS feasible. Compared to LC-MS, GC-MS generally provides superior separation efficiency, narrower chromatographic peaks, and highly reproducible retention times (rts), which can improve quantitative accuracy and method robustness. Furthermore, GC-MS instrumentation is often more accessible and cost-effective in many analytical laboratories, with lower operational costs and simpler maintenance requirements compared to high-resolution LC-MS systems. The technique can also provide excellent sensitivity for volatile or semi-volatile analytes such as ITCs. Indeed, GC-MS has been used for the determination of SF, particularly in broccoli and other Brassica products [11,16].

**Figure 2:**
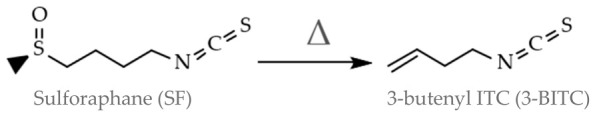
Thermal degradation of SF to 3-butenyl isothiocyanate (3-BITC).

Despite this, the thermal lability of SF can promote analyte degradation to 3-butenyl isothiocyanate (3-BITC) (Figure 2), thus negatively affecting quantitative accuracy and method reproducibility [16].

Chiang et al. (1998) [16] highlighted several critical aspects related to the GC determination of SF, demonstrating that chromatographic conditions and instrumental parameters significantly influence analyte stability and recovery. That study emphasized the importance of optimizing injection conditions and chromatographic settings to minimize thermal decomposition phenomena and improve analytical reliability. Although this work represented an important contribution to the development of GC methodologies for SF analysis, further investigations are still needed to better evaluate the influence of additional instrumental parameters that may affect analyte stability and chromatographic performance.

Based on these premises, this study aimed at evaluating the feasibility of GC-based methodologies for determining SF in dietary supplements. To this end, the effect of analytical parameters such as passage through the split/splitless injector, exposure to oven temperature during column transit and passage through the transfer line to the mass spectrometer on SF degradation to 3-BITC was investigated. The optimized method was then applied to the analysis of two commercial broccoli-based dietary supplements as a tool to predict their quality with an integrated approach combining HPLC profiling and quantification of GSLs with GC-MS determination of ITCs.

## 2. Results and Discussion

### 2.1. Analysis of Sulforaphane Thermal Degradation During the Gas Chromatographic Process

#### 2.1.1. Degradation During Column Elution

The results reported by Chiang et al. (1998) clearly [16] demonstrated that SF degrades to 3-BITC during GC analysis operated at high temperatures (250 °C) commonly used in conventional split/splitless GC injectors. Chiang et al. (1998) [16] further showed that the extent of degradation can be markedly reduced, from approximately 80% to 5% of the initial SF content, by adopting suitable modifications of instrumental GC conditions reducing the residence time of the analyte in the injector. This was accomplished by increasing the carrier gas flow rate at the beginning of the GC run (fast initial injection flow) in combination with the use of a liner characterized by a specific geometry. However, no investigation was undertaken to determine the injector temperature threshold at which the degradation begins. Moreover, the temperature above which SF becomes susceptible to thermal decomposition has not yet been clearly established.

**Figure 3:**
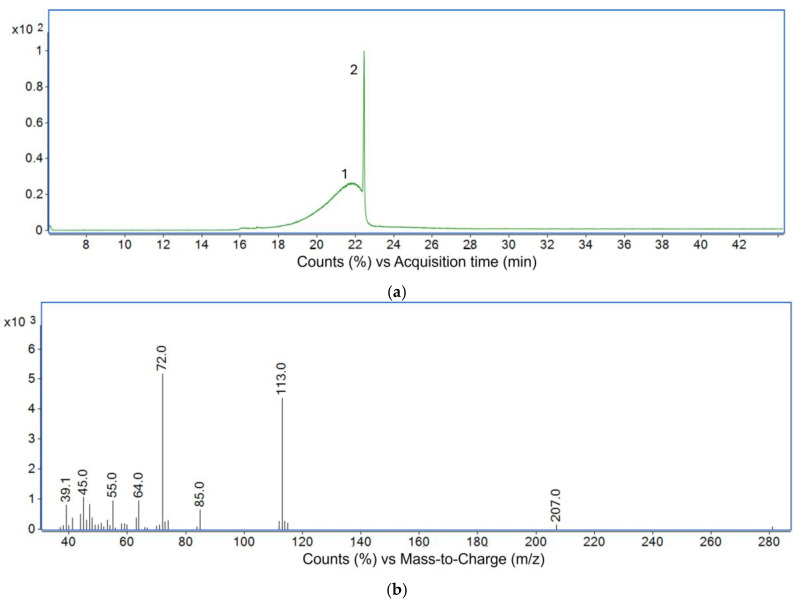
(**a**) Degradation of SF during elution through the GC column due to oven temperature 228 °C at elution time of SF (rt 22.60 min). Broad peak of 3-BITC (1) and peak of residual SF (2). GC conditions: GC column: DB5-MS UI, 60 m × 250 µm × 0.25 µm; injection at 150 °C, split ratio 250:1; temperature program: 70 to 240 °C (20 min) at 7 °C min^−1^; transfer line temperature: 250 °C. (**b**) MS spectrum detected for the broad peak eluting at 21.7 min and identified as 3-BITC.

It should first be noted that during chromatographic analysis, the stages at which the analyte may be exposed to relatively high temperatures are not limited to the passage through the injector but also include elution along the chromatographic column and the passage through the transfer line, typically maintained at a temperature higher than the maximum oven temperature. Indeed, during the preliminary experiments of the present study, a pronounced SF degradation was observed during its elution through the chromatographic column. This occurrence was evidenced by the appearance of a very broad chromatographic peak at an rt slightly preceding that of SF, identified as 3-BITC (Figure 3). The occurrence of this broad peak was consistent with the degradation of SF taking place during its transit through the column. A temperature of 150 °C was reached in the oven after 11.4 min, whereas at the elution time of SF from the column (22.6 min), it had reached 228 °C.

**Figure 4:**
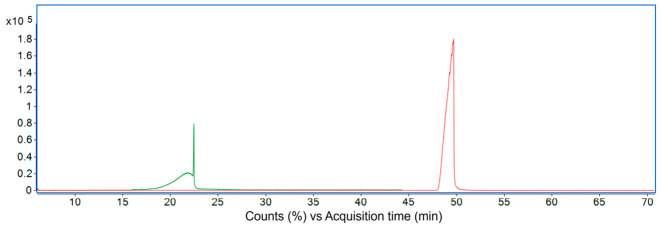
Removal of the cause of extensive thermal degradation by reducing the oven temperature to 150 °C until the elution of SF (red trace). GC conditions: GC column: DB5-MS UI, 60 m × 250 µm × 0.25 µm; injection at 150 °C, split ratio 250:1; temperature program: 70 °C (0 min), to 150 °C (60 min) at 10 °C min^−1^; transfer line temperature: 150 °C. Green trace: GC conditions as in Figure 3.

When the injector and transfer line temperatures were both maintained at relatively low values (150 °C), the broad degradation peaks were still detected, whereas no chromatographic peak corresponding to 3-BITC was observed at its expected rt. Further confirmation that these broad signals originated from the degradation of SF into 3-BITC during column passage was provided by their disappearance when elution was carried out under a temperature program with a constant final oven temperature of 150 °C. For example, upon application of the following temperature program (70 °C (0 min) to 150 °C (60 min) at 10 °C min^−1^), complete disappearance of the broad SF degradation peak was observed (Figure 4).

**Figure 5:**
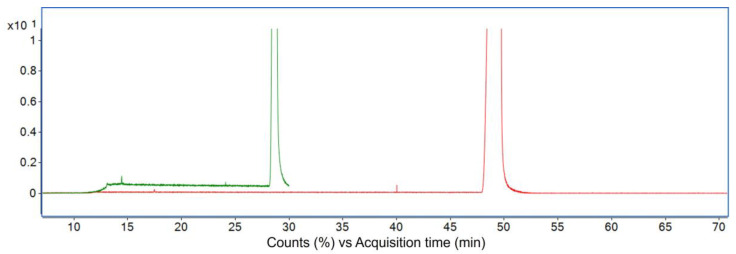
Effect of increasing the oven temperature from 150 °C to 170 °C (green trace). GC conditions: GC column: DB5-MS UI, 60 m × 250 µm × 0.25 µm; injection at 170 °C, split ratio 250:1; temperature program: 70 (0 min), to 170 °C (20 min) at 10 °C min^−1^; transfer line temperature: 170 °C (green trace). Red trace: GC conditions as in Figure 4.

Conversely, when the temperature program of −70 (0 min) to 170 °C (20 min) at 10 °C min^−1^ was applied, SF was exposed to a temperature of 170 °C in the column, and even at this temperature, a degradation effect became evident from the alteration of the chromatographic baseline at the corresponding rts (Figure 5). By applying the temperature program described above, a temperature of 150 °C is reached in the oven at min 8; then, starting from min 12, the onset of degradation of SF to 3-BITC is observed. The mass spectrum associated with this baseline disturbance confirmed the formation of 3-BITC.

This observation indicated that thermal degradation effects already began to be observed during exposure to 170 °C during column elution. Thus, under the chromatographic conditions reported in Figure 3, SF eluted from the column when the oven temperature had reached approximately 228 °C, thus causing extensive degradation of the compound while its chromatographic band was still located inside the column. In light of these observations, a temperature program was first established in order to ensure elution of SF from the column while the oven temperature remained below the threshold of 150 °C: 60 °C (1 min) to 150 (14 min) at 10 °C min^−1^ to 280 (10 min) at 20 °C min^−1^. It is noteworthy that this phenomenon was not reported by Chiang et al. (1998) [16]. Based on the chromatographic conditions described in their study, it can be estimated that SF eluted from the column at a temperature near 200 °C, virtually resulting in substantial degradation. However, no significant in-column degradation of the analyte was reported to occur in that previous study. This apparent discrepancy is merely speculative as no experimental investigation has been carried out in our work to directly replicate the published method.

#### 2.1.2. Degradation During Passage Through GC Injector and Transfer Line

In the previously mentioned study, the authors also showed that reducing the sample residence time inside the injector allowed the decrease in SF degradation from 80% of the initial content to approximately 5%. To that end, they used an injector with an internal diameter (i.d.) of 1.5 mm instead of 4.0 mm, and they increased the carrier gas flow rate from 1 mL min^−1^ to 3 mL min^−1^ at the beginning of the chromatographic run (fast initial injection flow) while maintaining splitless injection mode [16]. In the present study, the possibility of further reducing the degradation percentage was investigated by decreasing the injector temperature and the sample residence time by applying split injection rather than splitless injection. Indeed, SF concentration levels to be determined are generally compatible with splitless injection, and different split ratios can be applied while still preserving the required analytical sensitivity of the method.

To evaluate the effect of the split ratio on SF degradation, split ratios of 20:1, 50:1, and 100:1 were selected and compared with splitless injection. Secondly, different injector temperatures were tested, starting from 150 °C, a temperature at which no column degradation effect was observed, and subsequently increasing it to 200 °C, 225 °C, 240 °C and 250 °C. The latter temperature corresponded to the most commonly employed, under which, in combination with splitless injection, Chiang et al. (1998) [16] reported an SF degradation equal to 80% of its original content. It should be noted that Chiang et al. (1998) [16] considered lowering the injector temperature inappropriately, as this could result in material deposition on the column, impairing the separation performance. The percentages of SF degradation to 3-BITC observed under different combinations of split ratio and injector temperature values are shown in Table 1.

**Figure 6:**
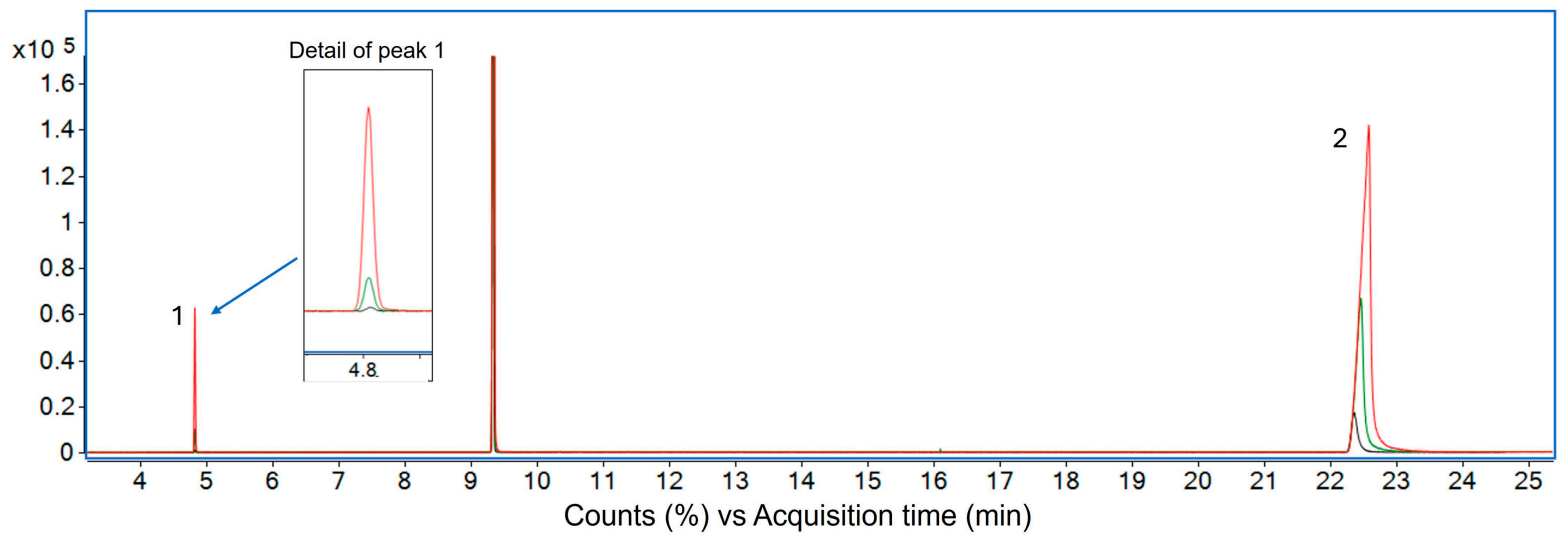
Increasing formation of the 3-BITC peak from the thermal degradation of SF (with injector temperature at 250 °C) for decreasing split ratios of 100:1 (black trace), 50:1 (green trace), and 20:1 (red trace). Peak 1: 3-BITC; peak 2: SF. Chromatographic conditions: GC column: HP5-MS UI, 30 m × 250 µm × 0.25 µm; injection at 250 °C; temperature program: 60 °C (1 min) to 150 (14 min) at 10 °C min^−1^ to 280 (10 min) at 20 °C min^−1^; transfer line temperature: 250 °C.

As shown in Figure 6, the peak height of 3-BITC decreased as the split ratio increased from 20:1 to 50:1 and 100:1. A concomitant decrease in the ratio between the peak heights of 3-BITC and SF was also observed.

As expected, both lowering the temperature and increasing the split ratio led to a reduction in the SF degradation percentage. Therefore, each factor independently contributed to mitigating the thermal degradation of SF. In the absence of splitting, a significant SF degradation of 4.3% was already observed at 200 °C. Conversely, when applying a split ratio of 20:1, SF degradation remained below 1% up to a temperature of 225 °C. In all these experiments, the 3-BITC peak eluted at the expected rt, confirming that degradation occurred in the injector and not during passage through the column, as observed above. Based on these results, a split ratio of 20:1 and an injector temperature of 225 °C were identified as the optimal analytical conditions, since they enabled minimization of SF degradation below 1% (95% confidence interval of the degradation percentage: 0.68–0.72%), while maintaining a relatively high injector temperature to avoid excessive deposition effects within the column. At the same time, the selected split ratio was not so high as to excessively reduce the sensitivity of the method.

After establishing the aforementioned conditions, the effect of the transfer line temperature on SF degradation was investigated. By setting the final oven temperature at 280 °C and the temperature of the transfer line at 290 °C, with the injector temperature at 150 °C and split ratio at 20:1, no formation of 3-BITC was observed. Accordingly, it can be concluded that exposure to high temperatures of the transfer line did not represent a significant cause of SF degradation.

### 2.2. Preliminary Studies for the Development of a Quantitative Method

After defining the operating conditions for the oven temperature program, injector settings (temperature and split ratio), and transfer line temperature, the selection of a suitable internal standard for manual injection determinations was undertaken. Tridecane and 2,6-dimethylphenyl ITC were evaluated, and the latter was selected due to the better repeatability of the peak area ratio (analyte/internal standard) (CV = 3.3%, *n* = 6 replicates).

For the preliminary development of a quantitative method, the linearity was assessed by constructing a five-point calibration curve over an SF concentration range of 75–625 μg mL^−1^, which was in the same order of magnitude as the one proposed by Chiang et al. (1998) [16]. Linear regression analysis of the area ratio (analyte/internal standard) versus SF concentration provided a correlation coefficient (R^2^) of 0.990. The limit of detection (LOD) and the limit of quantitation (LOQ) were estimated as 6.9 μg mL^−1^ and 23 μg mL^−1^, respectively. The LOD value was only slightly higher than that determined for the method proposed by Chiang et al. (1998) [16], confirming that the application of a 20:1 split ratio, instead of injection in splitless mode, did not compromise the sensitivity of the analytical method.

A notable limitation of GC-MS methods for SF determination is the scarcity of fully validated analytical procedures. Most published GC-MS methods focused on minimizing thermal degradation and demonstrating qualitative or semi-quantitative analysis, whereas validated performance characteristics such as LOQ and LOD are generally not reported. In contrast, numerous LC-MS/MS methods provide comprehensive validation data, including sensitivity, selectivity and robustness, thus making LC-MS/MS the preferred technique for routine quantitative analysis. Literature articles about SF determination from biological matrices with LC-MS/MS reported a LOD of 0.005 μM and an LOQ of 0.02 μM in rat plasma [17] and an LOQ of 7.8 nM in human plasma [18]. The authors of this last study also reported an accuracy, expressed as a bias of −2.7%, for SF at the LOQ, together with an intra-day precision (repeatability) of 8.61% RSD at the LOQ. More recently, Ali Redha et al. (2024) [13] developed an LC-MS method for SF quantification with a LOD of 1.3 ng mL^−1^ and an LOQ of 3.9 ng mL^−1^. Their method showed excellent linearity (R^2^ = 0.9963) and high selectivity, based on consistent rt and peak shape between standards and samples. Although the authors stated that accuracy (expressed as percentage bias) and precision were evaluated according to predefined acceptance criteria (±10%), the corresponding numerical validation results were not reported.

### 2.3. Analysis of Glucosinolates in Broccoli-Based Dietary Supplements

Two different broccoli-based dietary supplements were purchased online from two manufacturers in Italy (Table 2). Supplement S1 was labeled to contain a broccoli leaf dry extract standardized in 0.3% SF. The other supplement, S2, was claimed to contain a broccoli seed dry extract delivering stabilized 5% SF. As a first approach, we analyzed hydroalcoholic extracts of both supplements for GSL profiling and content determination by HPLC-PDA analysis of the corresponding desulfo-GSLs (dGSLs) according to the EU standard procedure ISO 9167:2019 [19].

#### 2.3.1. Supplement 1—Broccoli by Farmacia Legnani

**Figure 7:**
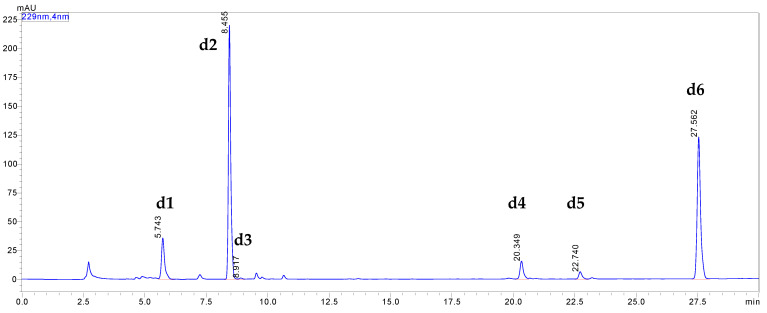
HLPC-PDA chromatogram of dGSLs obtained from the hydroalcoholic extract of the broccoli-based Supplement 1. Detection at 229 nm. Legend: (*Rs*)-3-(methylsulfinyl)propyl dGSL (d-glucoiberin, **d1**), (*Rs*)-4-(methylsulfinyl)butyl dGSL (d-glucoraphanin, **d2**), 2-propenyl dGSL (d-sinigrin, **d3**), indol-3-ylmethyl dGSL (d-glucobrassicin, **d4**), 4-methoxyindol-3-ylmethyl dGSL (d-4-methoxyglucobrassicin, **d5**), and 1-methoxyindol-3-ylmethyl dGSL (d-neoglucobrassicin, **d6**).

**Figure 8:**
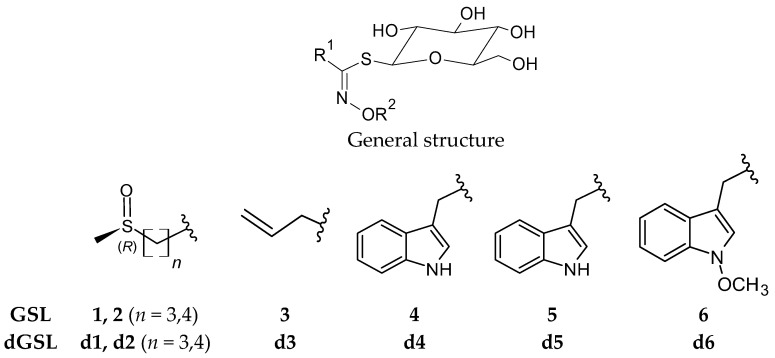
Structures of glucosinolates (GSLs) (R^2^ = SO_3_^−^) (**1**–**6**) identified and quantified in the broccoli-based Supplement 1 by HPLC-PDA analysis of the corresponding desulfo-glucosinolates (dGSLs) (R^2^ = H) (**d1**–**d6**). Legend: (*Rs*)-3-(methylsulfinyl)propyl GSL (glucoiberin, **1**), (*Rs*)-4-(methylsulfinyl)butyl GSL (glucoraphanin, **2**), 2-propenyl GSL (sinigrin, **3**), indol-3-ylmethyl GSL (glucobrassicin, **4**), 4-methoxyindol-3-ylmethyl GSL (4-methoxyglucobrassicin, **5**), and 1-methoxyindol-3-ylmethyl GSL (neoglucobrassicin, **6**).

The typical HPLC-PDA chromatogram obtained for S1 displayed five peaks identified according to the elution order as thiofunctionalyzed (*Rs*)-3-(methylsulfinyl)propyl dGSL (d-glucoiberin, **d1**) and (*Rs*)-4-(methylsulfinyl)butyl dGSL (d-glucoraphanin, **d2**), minor 2-propenyl dGSL (d-sinigrin, **d3**), and three indole dGSLs, indol-3-ylmethyl dGSL (d-glucobrassicin, **d4**), 4-methoxyindol-3-ylmethyl dGSL (d-4-methoxyglucobrassicin, **d5**) and 1-methoxyindol-3-ylmethyl dGSL (d-neoglucobrassicin, **d6**) (Figure 7 and Figure 8). The first three all originated from the methionine (Met) biosynthetic pathway, whereas the indole ones originated from tryptophan (Trp), according to the GSL classification based on the biosynthetic amino acid precursor [20].

The GSL pattern found in S1 was consistent with the typical GSL profile of broccoli leaf analyzed as a reference material in our lab and literature data [21], reflecting the description provided on the label about the vegetable ingredients used.

Individual and total GSL content quantified for S1 is summarized in Table 3. The most abundant GSL was glucoraphanin (**2**), representing 70% of total GSLs, followed by the homolog glucoiberin (**1**) and indole neoglucobrassicin (**6**). It is well known that broccoli seeds and sprouts display a predominance of sulfur-containing chain GSLs like glucoraphanin and glucoiberin, whereas relatively high indole GSLs are contained in broccoli at a later developmental stage [9,22]. Thus, the GSL composition assessed for S1 fits with the description of the vegetable material source. Previous studies reporting quantitative analysis of GSLs in brassica-based supplements are scarce, and data about supplements manufactured with broccoli leaf ingredients for comparison are not available [9,23]. However, total GSL content found in S1 was rather low compared to the range 1.0–35.8 mg cps^−1^ previously reported for 14 broccoli supplements mainly based on sprout or seed extracts [9], according to expectations based on the labeled broccoli leaf dry extract.

#### 2.3.2. Supplement 2—Rapha Myr+^®^ by Farmabarocco

No GSLs were detectable in samples of dietary supplement S2. The absence of GSLs led us to exclude the possibility that S2 comprised glucoraphanin and the active myrosinase enzyme, which is one of the commercial formulation strategies to provide SF; rather, it comprised a stabilized preparation of plant-derived SF. This occurrence matched the manufacturer’s description, claiming that the product contained stabilized SF without mentioning the precursor glucoraphanin. In a previous study, Tomasello et al. (2020) [24] investigated the anticancer activity of an aqueous extract Rapha Myr^®^ (Farmabarocco) supplement, demonstrating that the product is a promising chemotherapeutic agent for integrated cancer therapy of human astrocytoma. The Rapha Myr^®^ used in that study was reported as a novel blend of broccoli seed extract (*Brassica Oleracea* s.e., sulforaphane glucosinolate—that is, glucoraphanin-titer 11%) plus active myrosinase. Reasonably, glucoraphanin-based Rapha Myr^®^ was a different formulation than the SF-based Rapha Myr+^®^ evaluated in our study.

### 2.4. Hydrolysis of Broccoli-Based Dietary Supplements and Isothiocyanates Extraction

Hydrolysis experiments of the GSL-based supplement S1 were performed with or without incubation with exogenous commercial myrosinase, monitoring dGSL peak areas by HPLC-PDA analysis. Autolysis experiments demonstrated the absence of endogenous myrosinase activity in S1 because peak areas remained unchanged over time. Therefore, ITCs for GC-MS analysis were produced with the addition of commercial myrosinase followed by extraction with CH_2_Cl_2_.

Conversely, ITCs of supplement S2 were extracted directly, treating the capsule content with CH_2_Cl_2_.

### 2.5. GC-MS Analysis of Isothiocyanate Extracted from Broccoli-Based Dietary Supplements

**Figure 9:**
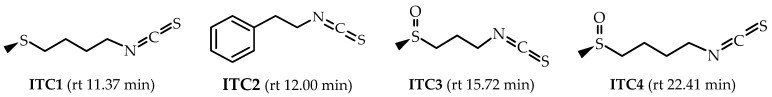
Structures of isothiocyanates (ITCs) issued from exogenous myrosinase hydrolysis of glucosinolates (GSLs) for supplement S1 and ITCs directly extracted from supplement S2. ITCs identified by GC-MS analysis are reported with retention time in order of elution. Legend: 4-(methylsulfanyl)butyl ITC (erucin, **ITC1**), 2-phenylethyl ITC (nasturtiin, **ITC2**), (*Rs*)-3-(methylsulfinyl)propyl ITC (iberin, **ITC3**), (*Rs*)-4-(methylsulfinyl)butyl ITC (sulforaphane, **ITC4**).

ITCs in CH_2_Cl_2_ extracts were analyzed by GC-MS using the method optimized in this study. ITC structures are illustrated in Figure 9, according to the elution order, whereas spectral data and content are summarized in Table 4.

#### 2.5.1. Supplement 1—Broccoli by Farmacia Legnani

SF (**ITC4**) was detected as the principal ITC, followed by its homolog iberin (**ITC3**) in S1 organic extract. These two sulfoxide ITCs resulted from exogenous myrosinase hydrolysis of GSLs, confirming the presence of intact glucoraphanin and glucoiberin in the supplement identified by HPLC-PDA analysis. Additionally, GC-MS analysis showed the presence of minor 2-phenylethyl ITC (nasturtiin, **ITC2**), whose unmodified precursor GSL was not revealed by HPLC. Only aliphatic GSLs accounted for conversion to ITCs; thus, indole GSLs are known to be hydrolyzed into highly unstable ITCs, which spontaneously undergo rapid rearrangements initially to alcohols and eventually to alcohol derivatives and condensation products [25].

**Table 4:** Free isothiocyanates (ITCs) identified by GC-MS analysis of dichloromethane extracts of two broccoli-based dietary supplements.

Isothiocyanates (ITCs)				S1	S2
peak #	Semisystematic name (*trivial name*)	Precursor GSL	MS spectral data (*m/z*) ^1^	Content (µg cps^−1^)	
ITC1	4-(methylsulfanyl)butyl ITC (*erucin*)	4-(methylsulfanyl)butyl GSL (*glucoerucin*)	**61**, 115, 161 (*M*^+^) Lit. ^2,3^	n.d.	27.80 ± 3.07
ITC2	2-phenylethyl ITC (*nasturtiin*)	2-phenylethyl GSL (*gluconasturtiin*)	**91**, 105, 163 (*M*^+^) Lit. ^2,4,5^	0.42 ± 0.03	n.d.
ITC3	(*Rs*)-3-(methylsulfinyl)propyl ITC (*iberin*)	(*Rs*)-3-(methylsulfinyl)propyl GSL (*glucoiberin*)	**72**, 101, 163 (*M*^+^) Lit. ^3,4^	1.72 ± 0.07	n.d.
ITC4	(*Rs*)-4-(methylsulfinyl)butyl ITC (*sulforaphane*)	(*Rs*)-4-(methylsulfinyl)butyl GSL (*glucoraphanin*)	**72**, 160, 177 (*M*^+^) Lit. ^3,4,6^	7.83 ± 0.21	38.24 ± 1.99
**Total**				9.97 ± 0.31	66.04 ± 5.06

^1^ Base ion in bold. Literature: ^2^ [26]; ^3^ [27]; ^4^ [28]; ^5^ [29]; ^6^ [16].

#### 2.5.2. Supplement 2—Rapha Myr+^®^

In S2 extracts, SF was detected as the main ITC together with its reduced form, 4- (methylsulfanyl)butyl ITC (erucin, **ITC1**). This ITC profile was consistent with broccoli seeds, which are a rich source of the precursor GSLs glucoraphanin and glucoerucin, thus fitting with the S2 label. It is therefore reasonable to speculate that broccoli seeds have been used as a formulation strategy to produce an SF-based preparation [10].

#### 2.5.3. Remarks About SF Evaluation in Both Supplements

The optimized GC-MS method allowed for reliable detection of free ITCs in dietary supplement extracts, minimizing thermal degradation of SF to 3-BITC. In earlier studies, the correct correlation of ITCs detected by GC-MS analysis with precursor GSLs in Brassicaceae vegetable materials was questioned. As a matter of fact, thermal degradation may lead to analytical artifacts showing ITC profiles that do not necessarily match the authentic ones resulting from the hydrolysis of precursor GSLs present in the vegetable material. Indeed, previous studies have reported thermal degradation of thiofunctionalized ITCs bearing a terminal sulfoxide moiety, namely SF homolog (*Rs*)-5-(methylsulfinyl)pentyl ITC (alyssin) and (*Rs*)-6-(methylsulfinyl)hexyl ITC (hesperin) into 4-pentenyl ITC and 5-hexenyl ITC [30,31,32]. The mechanistic pathway of thermal degradation of SF to 3-BITC possibly involves β-elimination of methanesulfenic acid, as suggested by Cedrowski et al. (2021) [33]. However, the proposed pathway is merely speculative as no further experimental investigations were carried out in our study.

It is noteworthy to mention that GC-MS analysis allowed only for the evaluation of free ITCs. As a matter of fact, ITCs are reactive molecules with high affinity for protein thiol functions. In fact, ITCs extracted from plant material may exist as free and as conjugates [22,25]. As a result, the ITC content determined by GC-MS may underestimate the ITC provided by the supplement intake, as a not-well-defined portion of protein-bound ITCs was not analytically considered. Total ITCs, free and protein-bound, are analyzed by the cyclocondensation assay with 1,2-benzenedithiol coupling to produce benzo [d]-1,3-dithiole-2-thione [22]. However, this determination was out of the scope of the present study, as we aimed at defining the supplement composition in terms of individual GSLs and/or ITCs. Therefore, the free SF content determined by GC-MS in our study was not compared to labeled SF (%) to avoid misleading conclusions. Overall, the presented study demonstrated the applicability of the set analytical approach, but it does not allow broader conclusions regarding the quality of broccoli-based supplements available on the market.

### 2.6. Study Limitations

The aim of the present study was not to provide a complete validation of a GC-MS analytical method for SF determination but rather to investigate and improve the understanding of its degradation during the gas chromatographic analysis process. Therefore, a complete method validation procedure was not performed; instead, the study aimed to provide preliminary elements supporting such a validation. Nevertheless, these preliminary findings confirmed the potential of the developed analytical approach, indicating the possibility of fully developing and validating, in the future, an analytical method with suitable characteristics for the analysis of SF-labeled dietary supplements.

## 3. Materials and Methods

### 3.1. Chemicals

#### 3.1.1. GC-MS Analysis

Pure standards used: D,L-sulforaphane (Toronto Research Chemicals Inc., Vaughan, ON, Canada), 3-BITC and 2,6-dimethylphenyl ITC (Santa Cruz Biotechnology, Dallas, TX, USA), tridecane (Merck Italia, Milan, Italy). Solvents: CH_2_Cl_2_ and GC-grade CH_2_Cl_2_ were purchased from Carlo Erba Reagents (Milan, Italy).

#### 3.1.2. HPLC-PDA Analysis of Desulfo-Glucosinolates

DEAE Sephadex A-25 resin was purchased from GE Healthcare (Freiburg, Germany). Analytical-standard sinigrin (prop-2-enyl glucosinolate), myrosinase (*β*-thioglucoside glucohydrolase; EC 3.2.1.147) from *Sinapis alba* (white mustard seed), and sulfatase from *Helix pomatia* (EC 3.1.6.1) were obtained from Sigma-Aldrich (St. Louis, MO, USA). HPLC-grade water and CH_3_CN were from Pancreac Quimica SLU (Barcelona, Spain) and Riedel-de Haën (Seelze, Germany), respectively.

### 3.2. Broccoli-Based Dietary Supplements

Two commercial broccoli-based dietary supplements were purchased and analyzed. The numbering used and the composition of both supplements declared by the manufacturer/supplier are summarized in Table 2.

### 3.3. Glucosinolate Analysis of Broccoli-Based Dietary Supplements

GSLs were analyzed according to the EU standard procedure ISO 9167:2019 [19]. Content samples of each supplement were extracted twice with boiling EtOH-H_2_O (7:3 *v*/*v*, 5 mL each cps) for 3 min at 80 °C (S1: ca. 300 mg/10 mL; S2: ca. 440 mg/5 mL) and then centrifuged with a 5810R centrifuge (Eppendorf AG, Hamburg, Germany) at 9000× *g* rpm for 10 min at 10 °C. Supernatants were combined, and each extract (1 mL) was loaded onto a mini-column filled with DEAE-Sephadex A-25 anion-exchange resin (0.6 mL) conditioned with 25 mM sodium acetate buffer (pH 5.6). After washing with the same buffer (3 mL), sulfatase purified by fractional precipitation (200 μL) [34] was loaded onto the mini-column and left for overnight reaction. Extraction and desulfation were performed in duplicate. DGSLs were then eluted with ultra-pure H_2_O (3 mL) and analyzed on an HPLC-PDA LC-40 model (Shimadzu, Kyoto, Japan) system equipped with an Avantor ACE 5 C18 column (250 × 4.6 mm, 4 μm particle size) (VWR, Darmstadt, Germany), thermostated at 30 °C, and having a PDA detector. The chromatography was performed at a flow rate of 1 mL min^−1^, eluting with a gradient of H_2_O (A) and CH_3_CN (B) with the following program: 1 min 1% B; 30 min linear gradient up to 30% B; 3 min linear gradient down to 1% B. DGSLs were detected by absorbance monitoring at 229 nm. Identification of the peaks was performed based on rts and UV spectra of standards available in our library. The GSL amount was quantified by using a calibration curve of pure d-sinigrin solution (range from 0.06 to 1.93 mM) and the relative proportionality factor (RPF) of each individual dGSL [35,36]. RPF values for quantification were as follows: 1.07 for d-glucoiberin and d-glucoraphanin, 0.29 for d-glucobrassicin, 0.20 for d-4-methoxyglucobrassicin and 0.25 for d-neoglucobrassicin.

### 3.4. Autolysis of Broccoli-Based Dietary Supplement S1 and Monitoring of Glucosinolates

The contents of 2 capsules (ca. 300 mg) of dietary supplement S1 were mixed at room temperature for 30 min with HPLC-grade water (5 mL, pH 6.5) by vortexing for 1 min every 5 min. The resulting mixture was centrifuged with a 5810R centrifuge (Eppendorf AG, Hamburg, Germany) at 9000× *g* rpm for 10 min at 10 °C. The supernatant was collected and stored at −20 °C until analysis. The water solution (1 mL) was analyzed for GSL monitoring by HPLC-PDA as described in the previous subsection.

### 3.5. Exogenous Myrosinase Hydrolysis of Broccoli-Based Dietary Supplement S1 for GC-MS Analysis

The contents of 2 capsules (ca. 300 mg) of dietary supplement S1 were placed in a screw-capped test tube, mixed with distilled water (5 mL, neutral pH), and vortexed to extract GSLs. The resulting mixture was centrifuged at 5000 rpm for 10 min at 20 °C, and the supernatant was collected. ITCs were produced by enzymatic hydrolysis of GSLs via addition of commercial myrosinase (5 U mL^−1^, 200 μL) to the aqueous solution. One myrosinase unit was defined as the amount of enzyme able to hydrolyze 1 μmol min^−1^ of sinigrin at neutral pH and 37 °C. After 30 min of stirring at room temperature, the resulting ITCs were extracted with CH_2_Cl_2_ (0.5 mL), filtered through a 0.22 μm PTFE filter, and stored at −27 °C prior to GC analysis.

### 3.6. Isothiocyanate Extraction from Broccoli-Based Dietary Supplement S2

The content of one capsule (ca. 440 mg) of the dietary supplement S2 was mixed with CH_2_Cl_2_ (5 mL) and dissolved by ultrasound using an Elmasonic S 100 H ultrasound bath (Elma Schmidbauer GmbH, Singen, Germany). Subsequently, the organic solution was filtered through a 0.22 μm PTFE filter and stored at −27 °C prior to GC analysis.

### 3.7. Gas Chromatography–Mass Spectrometry

All GC-MS analyses were performed on an Agilent 6890 GC 5973N MS system equipped with a quadrupole mass filter for mass spectrometric detection (Agilent Technologies, Palo Alto, CA, USA). Various combinations of instrumental settings were applied during the progress of the study. The conditions employed in the different experiments are reported in the Results section. GC separation was achieved on a DB-5MS UI column (60 m × 250 μm × 0.25 μm) for preliminary tests and on a HP-5MS UI column (30 m × 250 μm × 0.25 μm) for the following experiments. Both GC columns were manufactured by J&W, Agilent Technologies (Palo Alto, CA). GC injection temperature and split condition (split ratio or no split applied), oven temperature program, and transfer line temperature were changed from time to time. A constant flow of He carrier gas was set at 1.5 mL min^−1^, corresponding to a linear average velocity of 31.9 cm s^−1^ (on DB-5MS) and 44.8 cm s^−1^ (on HP-5MS), respectively. The MS detector operated in the electronic ionization mode at 70 eV; source and quadrupole temperatures were set at 230 and 150 °C, respectively. Detection was performed in the full scan mode, over the mass range 30–300 amu. Identification of SF and 3-BITC in the analyzed extracts was carried out by comparing mass spectra and linear retention indices (LRIs) with those obtained from authentic standards. The extent of SF degradation to 3-BITC was estimated as the percentage ratio of the 3-BITC peak area to the sum of the peak areas of the two compounds.

### 3.8. Dilution and Standard Solutions

Thermal degradation experiments of SF were carried out using a standard solution of SF in CH_2_Cl_2_ at a concentration of 875 μg mL^−1^. Standard solutions for building a calibration curve contained SF from 75 to 625 μg mL^−1^ and the internal standard at 685 μg mL^−1^ in CH_2_Cl_2_.

### 3.9. GC-MS Analysis of Free Isothiocyanates from Broccoli-Based Dietary Supplements

For the analysis of ITC-containing extracts of the two dietary supplements, the previously described optimized method was applied using the following analytical conditions: GC column HP-5MS; injector temperature at 225 °C and split ratio at 20:1; oven temperature program: 60 °C (1 min), to 150 (14 min) at 10 °C min^−1^ to 280 (10 min) at 20 °C min^−1^; constant flow of He carrier gas was set at 1.5 mL min^−1^; MS detector settings as reported in Section 3.7. Identification of SF was carried out as specified above. Additional ITCs were identified by matching mass spectra and LRIs with data from the available NIST/EPA/NIH Mass Spectra Library 2017 and by comparison with literature sources.

### 3.10. Statistical Analysis

Results obtained on percentage of SF degradation were subjected to one-way ANOVA and Tukey’s HSD post hoc test for multiple comparisons (*p* < 0.05) or to Student’s t-test to assess statistical significance of differences observed between the considered GC conditions. Statistical analyses were carried out using XLStat software (version 2025.02.0; Addinsoft, New York, NY, USA).

## 4. Conclusions

This study demonstrated that SF is highly susceptible to thermal degradation to 3-BITC during GC-MS analysis and that chromatographic conditions play a crucial role in preserving its integrity throughout the analytical process. Optimization of the chromatographic parameters, including setting the injector temperature at 225 °C, employing a split ratio of 20:1, and maintaining the oven temperature below 150 °C until SF elution, reduced SF degradation to 3-BITC to less than 1%.

Two broccoli-based dietary supplements were examined through an integrated analytical approach including the official EU ISO 9167:2019 HPLC-PDA analysis of GSLs and the presented optimized GC-MS method for a reliable assessment of released free ITCs.

Results demonstrated two different compositions for the products examined. One supplement was glucoraphanin-based without active myrosinase, whereas the other one was formulated with stabilized SF. Qualitatively, results reflected the broccoli ingredient label, which was declared by the manufacturer in both cases.

In conclusion, the optimized method represents a valuable analytical tool for quality control purposes. Furthermore, when combined with GSLs analysis, it provides a more comprehensive evaluation of supplement composition and labeling accuracy, contributing to improved characterization of glucoraphanin or SF-containing ingredients for the supplement industry.

## Figures and Tables

**Table 1:** Percentages of degradation of sulforaphane (SF) to 3-butenyl isothiocyanate (3-BITC) under different GC injector temperature and split ratio conditions, using an oven program that ensured the elution of SF at a temperature not exceeding 150 °C. Data are reported as mean values and standard deviations obtained by 3 replicate injections. *p*-values are obtained by ANOVA or by Student’s t-test when only two determinations were to be compared. Different uppercase letters within a row indicate significant differences (according to Tukey’s test, at *p* > 0.05). Different lowercase letters within a column indicate significant differences (according to Tukey’s test, at *p* > 0.05).

GC Injector Temperature	Split Ratio				
	Splitless	20:1	50:1	100:1	*p*
150 °C	n.d. ^1^	n.d.	- ^2^	-	-
200 °C	4.39 ± 0.25	0.11 ± 0.02 d	n.d.	-	0.0011
225 °C	19.40 ± 1.20 A	0.70 ± 0.01 B, c	0.27 ± 0.02 B, c	n.d.	<0.0001
240 °C	>35	2.15 ± 0.20 A, b	0.86 ± 0.05 B, b	0.42 ± 0.02 C	<0.0001
250 °C	>60	4.29 ± 0.16 A, a	2.03 ± 0.05 B, a	0.97 ± 0.08 C	<0.0001
*p*	0.0015	<0.0001	<0.0001	0.0055	

^1^ n.d.: no 3-BITC detected, meaning no degradation of SF. ^2^ For each injector temperature value, when no degradation of SF was observed, no experimental determinations were carried out at higher split ratios.

**Table 2:** Data for the two commercial broccoli-based dietary supplements purchased and analyzed.

No.	Brand Name	Manufacturer/Supplier	Form	Broccoli Dry Extract	Extract (mg cps^−1^)	SF ^1^ (µg cps^−1^)
S1	Broccoli	Farmacia Legnani, Milano, Italy	Cps ^2^	*Brassica oleracea* L. var gemm (leaf)	80	240
S2	Rapha Myr+^®^	Farmabarocco, Ragusa, Italy	cps	*Brassica oleracea* L. var. italica (seed)	100	5000

^1^ Labeled SF content; ^2^ capsules.

**Table 3:** Individual and total glucosinolate (GSL) content assessed in the broccoli-based Supplement 1 by HPLC-PDA analysis of the corresponding desulfo-glucosinolate (dGSLs).

Glucosinolate (GSL)			Supplement 1 Content		
#	Class ^1^	Semisystematic name (*trivial name*)	µmol cps^−1^	µg cps^−1^	% on total GSLs
**1**	Met	(*Rs*)-3-(Methylsulfinyl)propyl GSL (*glucoiberin*)	0.16 ± 0.01 ^2^	65.81 ± 3.19	14.6
**2**	Met	(*Rs*)-4-(Methylsulfinyl)butyl GSL (*glucoraphanin*)	0.72 ± 0.04	316.38 ± 15.43	70.3
**3**	Met	2-propenyl GSL (*sinigrin*)	>0.01	1.26 ± 0.31	0.3
**4**	Trp	indol-3-ylmethyl GSL (*glucobrassicin*)	0.03 ± 0.00	13.31 ± 0.05	3.0
**5**	Trp	4-methoxyindol-3-yl GSL (*4-methoxy glucobrassicin*)	0.01 ± 0.00	4.15 ± 0.24	0.9
**6**	Trp	1-methoxyindol-3-ylmethyl GSL (*neoglucobrassicin*)	0.10 ± 0.00	48.99 ± 1.86	10.9
**Total**			1.02 ± 0.05	449.90 ± 21.07	100

^1^ GSL classification on the basis of the biosynthetic amino acid precursor; ^2^ The data represent the mean ± SD of two replicated experiments with 2 samples analyzed per replicate (*n* = 4).

## Data Availability

Data are contained within the article.

## Abbreviations

The following abbreviations are used in this manuscript:

SF	Sulforaphane
GSL	Glucosinolate
ITC	Isothiocyanate
DGSL	Desulfo-glucosinolate
3-BITC	3-Butenyl isothiocyanate
LRI	Linear retention index
rt	Retention time
